# Supplementary material for: A multiplexed, single-cell sequencing screen identifies compounds that increase neurogenic reprogramming of murine Muller glia
Source: eLife. 2024 Dec 12;12:RP92091. doi: 10.7554/eLife.92091 (PMC11637464; doi:10.7554/eLife.92091)
Supplement: Supplementary file 1. [file elife-92091-supp1.docx]

**Supplementary File 1**

A multiplexed, single-cell sequencing screen identifies compounds that increase neurogenic reprogramming of murine Muller glia.

Amy Tresenrider^1^, Marcus Hooper^2^, Levi Todd^2^, Faith Kierney^2^, Nicolai Blasdel^2^, Cole Trapnell^*,1,3,4^, Thomas A. Reh*^,#2^

Thomas A. Reh and Cole Trapnell

Email: [tomreh@uw.edu,](about:blank) coletrap@uw.edu

**Supplementary file 1a.** Marker genes used for cell type annotation.

| **Experiment** | **Cell Type** | **Markers** | | | | | | |
| --- | --- | --- | --- | --- | --- | --- | --- | --- |
| **Tc/pulse** | MG | *Aqp4* |  |  |  |  |  |  |
| **Tc/pulse** | ProL | *Ascl1* | *↓Aqp4* | *Hes6* | *Dll1* | *Dll3* |  |  |
| **Tc/pulse** | Transition | *high Ascl1* | *↓Aqp4* | *↑Snap25* | *↑Dcx* | *Hes6* | *Dll1* | *Dll3* |
| **Tc/pulse** | NeuPre | *Snap25* | *Dcx* | *Gap43* | *Hes6* | *Dll3* | *Elavl3* |  |
| **Tc/pulse** | BP | *Otx2* | *Cabp5* | *↑Snap25* | *↓Hes6* | *↓Dll3* | *Trpm1* | *Grm6* |
| **Tc/pulse** | Astrocyte | *Gfap* | *Pax2* |  |  |  |  |  |
| **Tc/pulse** | Microglia | *Spp1* | *P2ry12* |  |  |  |  |  |
| **Tc/pulse** | FibroL | *Fn1* | *Lox* | *Col4a1* |  |  |  |  |
| **Tc/pulse** | Unknown |  |  |  |  |  |  |  |
| **Screen_all** | Reprogramming | *Vsx2* | *Aqp4* | *Rlbp1* |  |  |  |  |
| **Screen_all** | Bipolar | *Otx2* | *Cabp5* | *↑Snap25* | *↓Hes6* | *↓Dll3* | *Trpm1* | *Grm6* |
| **Screen_all** | Astrocytes | *Gfap* | *Pax2* |  |  |  |  |  |
| **Screen_all** | Microglia | *Spp1* | *P2ry12* |  |  |  |  |  |
| **Screen_all** | FibroL | *Fn1* | *Lox* | *Col4a1* |  |  |  |  |
| **Screen_all** | Astrocyte-derived neuron | *Gap43* | *Dcx* | *Snap25* | *↓Vsx2* |  |  |  |
| **Screen_all** | Proliferating | *Mki67* | *Top2a* | *Col4a1* | *Fn1* | *Lox* | *Vsx2* | *Aqp4* |
| **Screen_mg** | MG | *Aqp4* | *Apoe* |  |  |  |  |  |
| **Screen_mg** | ProL (early) | *Aqp4* | *Gfap* | *Apoe* | *Hes5* | *Fosb* | *Ascl1* |  |
| **Screen_mg** | ProL (late) | *Ascl1* | *Fn1* | *Hes5* |  |  |  |  |
| **Screen_mg** | Transition | *Ascl1* | *Gfap* | *Snap25* |  |  |  |  |
| **Screen_mg** | NeuPre | *Gap43* | *Snap25* | *Dcx* |  |  |  |  |
| **Screen_mg** | BP | *Snap25* | *Vsx2* | *Cabp5* | *Otx2* | *Grik1* |  |  |
| **Screen_mg** | ER stressed | *Hspb1* | *Trib3* |  |  |  |  |  |
| **Screen_mg** | Immune reactive | *Pik3ap1* | *Mpeg1* |  |  |  |  |  |
| **Screen_mg** | Undefined neuron | *Otx1* | *Neto1* |  |  |  |  |  |
| **Screen_mg** | Unknown | *Slc5a5* | *Perp* |  |  |  |  |  |
| **In_vivo** | MG | *Aqp4* | *Rlbp1* |  |  |  |  |  |
| **In_vivo** | Transition | *Aqp4* | *Rlbp1* | *Ascl1* |  |  |  |  |
| **In_vivo** | NeuPre | *Otx2* | *Nrxn3* |  |  |  |  |  |
| **In_vivo** | Bipolar | *Snap25* | *Cabp5* | *Syt1* | *Nrxn3* |  |  |  |
| **In_vivo** | Cones | *ARR3* | *SYT1* | *SNAP25* |  |  |  |  |
| **In_vivo** | Rods and Proliferating Cells | *Rho* | *Nrl* | *Mki67* | *Top2a* |  |  |  |
| **In_vivo** | Astrocyte | *Pax2* | *Gfap* |  |  |  |  |  |
| **In_vivo** | Microglia | *Tmem119* | *P2ry12* | *Spp1* |  |  |  |  |
